# Bone Response to Dietary Co-Enrichment with Powdered Whole Grape and Probiotics

**DOI:** 10.3390/nu10020146

**Published:** 2018-01-29

**Authors:** Cynthia Blanton

**Affiliations:** Nutrition and Dietetic Programs, Idaho State University, 921 S 8th Ave., Pocatello, ID 83209, USA; blancynt@isu.edu; Tel.: +1-208-282-3953

**Keywords:** grapes, probiotics, bone, mice, aging

## Abstract

Nutrition is a primary modifiable determinant of chronic noncommunicable disease, including osteoporosis. An etiology of osteoporosis is the stimulation of bone-resorbing osteoclasts by reactive oxygen species (ROS). Dietary polyphenols and probiotics demonstrate protective effects on bone that are associated with reduced ROS formation and suppressed osteoclast activity. This study tested the effect of dietary enrichment with powdered whole grape and probiotics (composed of equal parts *Bifidobacterium bifidum, B. breve, Lactobacillus casei, L. plantarum*, and *L. bulgaricus*) on bone microarchitecture in a mouse model of age-related osteoporosis. Groups (*n* = 7 each) of 10-month-old male mice were fed one of six diets for 6 months: 10% grape powder with sugar corrected to 20%; 20% grape powder; 1% probiotic with sugar corrected to 20%; 10% grape powder + 1% probiotic with sugar corrected to 20%; 20% grape powder + 1% probiotic; 20% sugar control. Femur, tibia and 4th lumbar vertebrae from 10-month-old mice served as comparator baseline samples. Bone microarchitecture was measured by micro-computed tomography and compared across diet groups using analysis of variance. Aging exerted a significant effect on tibia metaphysis trabecular bone, with baseline 10-month-old mice having significantly higher bone volume/total volume (BV/TV) and trabecular number measurements and lower trabecular spacing measurements than all 16-month-old groups (*p <* 0.001). Neither grape nor probiotic enrichment significantly improved bone microarchitecture during aging compared to control diet. The combination of 20% grape + 1% probiotic exerted detrimental effects on tibia metaphysis BV/TV compared to 10% grape + 1% probiotic, and trabecular number and trabecular spacing compared to 10% grape + 1% probiotic, 1% probiotic and control groups (*p <* 0.05). Femur metaphysis trabecular bone displayed less pronounced aging effects than tibia bone, but also showed detrimental effects of the 20% grape + 1% probiotic vs. most other diets for BV/TV, trabecular number, trabecular spacing and trabecular pattern factor (*p <* 0.05). Tibia and femur diaphysis cortical bone (cortical wall thickness and medullary area) displayed neither aging nor diet effects (*p* > 0.05). Vertebrae bone showed age-related deterioration in trabecular thickness and trabecular spacing and a trend toward preservation of trabecular thickness by grape and/or probiotic enrichment (*p <* 0.05). These findings demonstrate no benefit to bone of combined compared to independent supplementation with probiotics or whole grape powder and even suggest an interference of co-ingestion.

## 1. Introduction

Bone protection is paramount as people are living longer and must maintain optimal skeletal health into advanced age. A 2015 analysis of US National Health and Nutrition Examination Survey data reports the prevalence of low bone mass as 48% and osteoporosis as 16% in adults over age 65 years [1]. Despite this high rate, osteoporosis is an underdiagnosed and undertreated disease, often not recognized until bone fracture occurs [2,3,4]. Strikingly, up to 50% of vertebral fractures incidentally discovered by radiography occur in patients without prior diagnosis of osteoporosis [5]. By 2025, an expected three million osteoporosis-related fractures will cost the US health care system $25 billion [6]. Prevention is key to reducing the personal and societal burdens of osteoporosis.

Nutrition intervention offers an inexpensive, readily accessible, preventive strategy against osteoporosis that is most effective when adopted early in life [7]. Observational population studies showing bone-protective effects of fruit consumption [8,9,10,11,12,13,14,15,16,17,18] have been supported by mechanistic animal and cellular investigations demonstrating an inhibitory effect of fruit polyphenols on osteoclast formation and activity [19,20,21,22,23,24]. For example, in a cross-sectional study of 3089 Chinese men and women of age 40–75 years old, Qui et al. [8] found significantly higher total and site-specific bone mass density (BMD) in participants consuming the highest vs. lowest tertile of fruit. This effect was consistent for femoral neck BMD across five of six subcategories of fruit, including the grapes/litchi/longan subcategory. From the perspective of fracture risk, Byberg et al. [10] examined the effect of fruit and vegetable intake over a mean 14.2 years in a cohort of 75,591 Swedish men and women. This group reported a 1.39 hazard ratio for hip fracture in people consuming zero vs. 3 servings of fruit per day. Welch et al. [17] focused on the effect of dietary flavonoids, a group of plant compounds with biologic activity, on bone density in 3160 women from the TwinsUK cohort. Results showed a positive association between total flavonoid intake and higher spine BMD. Further, the strongest beneficial effect on hip and spine BMD was seen for high intake of anthocyanins, a subclasss of flavonoids that are concentrated in grapes. 

Grapes are one of the most concentrated sources of dietary polyphenols [25] and they have attracted considerable attention as a nutrition intervention against degenerative disease, including osteoporosis. Grape intake is thought to protect bone by blocking inflammatory molecules [26] that stimulate osteoclasts (bone-degrading cells) [20,21,22] and by promoting activity of osteoblasts (bone-building cells) [23,24,27,28]. This mechanism is consistent with the concept of age-related bone loss being largely due to chronic low-grade inflammation [29,30,31]. Grape polyphenol isolates, such as resveratrol, have been widely used to investigate their effect on bone [18]. Resveratrol dietary supplementation demonstrates bone-protective effects in multiple experimental models including obese men at high risk of bone loss [32], mice exposed to oxidative stress [33], ovariectomized rats (a model of post-menopausal osteoporosis) [34], rats with periodontal bone disease [35], and tail-suspended rats [36]. Although whole grape powder is more representative of a typical diet than polyphenol extracts, a grape-enriched diet has been tested in only one known study of bone health. Hohman et al. [37] showed significantly greater femur thickness and strength in ovariectomized rats fed a 25% grape vs. control diet for eight weeks. Further study of the bone response to whole grape and grape powder consumption is needed to inform dietary recommendations specific to bone preservation. 

A novel strategy to enhance the bone-protective effects of grape consumption is to increase the production of bioactive grape polyphenol metabolites in the gastrointestinal tract. Intestinal bacteria (microbiota) metabolize dietary polyphenols to compounds that are absorbed into the body and exert beneficial biologic effects [38,39,40]. Bacterial species that metabolize polyphenols to bioactive compounds have been identified and among these are species used as dietary probiotics. Probiotics are health-promoting bacteria added to foods such as yogurt that colonize the intestine [41] and transform undigested food components into biologically active compounds [40,41,42]. Research demonstrates that the most commonly consumed probiotic bacteria produce bioactive secondary metabolites from polyphenol-rich fruit [43,44,45,46], and mouse models indicate that these secondary metabolites of polyphenols augment the health benefits of polyphenol-rich food consumption, producing a synergistic effect [42,47,48]. The purpose of this study was to test the hypothesis that adding probiotics to a grape-enriched diet would augment the bone-protective effects of grape consumption. 

## 2. Methods

### 2.1. Experimental Design

The experiment was performed in strict accordance with the Guide for the Care and Use of Laboratory Animals prepared by the National Institutes of Health. The use of animals and the study protocol were approved by the Idaho State University Animal Care and Use Committee on 21 July 2016 under protocol number 743. Male 8-month-old ND4 Swiss Webster retired breeder mice were purchased from Envigo, Indianapolis, IN and were housed individually in one environmentally controlled room under a 12-h light-dark cycle and fed standard chow for two months. At age 10 months, a group (*n* = 6) of mice was used to provide bones for baseline reference measurements to which age- and diet-related changes in 16-month-old mice were compared. The remaining groups (*n* = 7) were fed one of six diets for 6 months (to age 16 months): 10% grape powder with sugar corrected to 20%; 20% grape powder; 1% probiotic with sugar corrected to 20%; 10% grape powder + 1% probiotic with sugar corrected to 20%; 20% grape powder + 1% probiotic; and 20% sugar control. 

### 2.2. Diets

Isonitrogenous diets were created based on a commercially available rodent chow (AIN-93M) modified with freeze-dried grape powder made from whole grapes and probiotics, (Table 1). Grape powder was provided by the California Table Grape Commission. The 20% grape powder concentration is similar to that used in three rodent studies of bone response using grape [37] or plum [49,50] powder. The 20% grape dose equates to a fresh grape intake of 4 servings per day in a 60-kg human [51]. The 10% grape powder diet was included to test for bone effects from a smaller dose. 

The probiotic was a mixture of equal parts *Bifidobacterium bifidum*, *B. breve, Lactobacillus casei*, *L. plantarum*, and *L. bulgaricus* (Nutraceutix^®^, Redmond, OR, USA) at a concentration of 10^11^ cfu/g. These strains were selected because they demonstrate growth and polyphenol metabolite production when cultured in polyphenol medium [44,45,46,52,53]. The 1% probiotic concentration provided 1.0 × 10^8^ cfu/g diet, or 5 × 10^8^ cfu/day for a mouse consuming 5 g diet/day, a dose effective in producing bone improvements in mice in our laboratory’s previous study [54]. Fresh batches of diet were prepared monthly at the same time, in the sequence of control, grape, grape + probiotic, and probiotic to minimize the risk of probiotic transfer to non-probiotic diets. Diets were stored frozen and replenished in food dishes daily to ensure viability of probiotics consumed. Our laboratory has previously demonstrated good stability/viability of probiotics mixed into powdered diet [55,56].

Mouse body weight and food intake were measured weekly. At the conclusion of the feeding experiment, mice were euthanized using carbon dioxide and femur, tibia and 4th lumbar spine vertebra were excised. Bones were cleaned of muscle, fixed in 10% formalin for 48 h, and stored in 70% ethanol at 4 °C until micro-computed tomography scanning.

### 2.3. Micro-Computed Tomography (µCT) Scanning of Bones

High-resolution µCT scans were performed at the University of Utah Small Animal Imaging Core Facility. Bones were scanned on a Siemens Inveon tri-modality (CT/PET/SPECT) system (Siemens AG, Munich, Germany) by a trained technician dedicated to the project. The energy settings used were 150 uA/80 kEv, with an exposure time of 2300 ms. The scans used a full rotation step-and-shoot acquisition with 600 total projections. The reconstructions from the acquired projections resulted in images with an isotropic spatial resolution of 20.49 µm with a matrix size of 1024 × 1024 × 3968.

One operator performed all of the analysis. Reconstructed images were loaded into Siemens Inveon Research Workstation or IRW (Siemens AG, Munich, Germany) software and checked for artifacts, such as motion artifacts and beam hardening effects. Once the reconstructed image quality was deemed acceptable, the desired bone regions (femur, tibia and vertebrae) were cropped and analyzed separately as follows. For the femur, the medial notch superior to the condyles was used as a mean to rotate the femur such that the condyles aligned with the horizontal axis. The tibia was rotated such that the flat posterior side was aligned with the horizontal axis whereas the vertebrae was aligned such that its anterior side was aligned with the horizontal axis. For cortical bone analyses, the femur and tibia were analyzed at the 50% mid-shaft whereas the vertebra was analyzed at the 50% mid-vertebrae region. For the data to reflect the area, a range of 21 slices (±10 slices from the 50% slice) was segmented. Trabecular bone analyses for the femur were performed in the distal femoral metaphysis region defined at 0.1 mm proximal to the distal femoral growth plate extending 1 mm towards the mid diaphysis. Tibia trabecular analyses were performed in the proximal tibia metaphysis defined at 0.1 mm distal to the growth plate of the proximal tibia extending 1 mm towards the diaphysis. Two additional regions were analyzed for the vertebrae, namely the regions at the 25% and 75%, for these additional ranges of interest (ROIs), a range of 15 slices was chosen however the bone morphometric (including the trabecular measurements) results were averaged to accurately reflect the entire vertebra. Measurement for each of the three ROIs was based on the total length of the vertebra measured from the cranial end following orientation. For the purposes of segmentation, a thresholding value based on the image volume histogram that selected 65% of the brightest of pixels was used. This value clearly differentiated the cortical bone from the trabecular bone and bone marrow and was kept consistent from specimen to specimen. 

Once each region was segmented, the Siemens IRW software (version 4.2, Munich, Germany) calculated bone morphometric measurements which included the ratio of total trabecular bone volume to total tissue (bone + marrow) volume (BV/TV, in %); the ratio of trabecular bone surface area to trabecular total bone volume (BS/TV, in µm^−1^), representing the proportion of bone surface available for remodeling; trabecular thickness (Tb.Th, in µm); trabecular number (Tb.N, in µm^−1^), in which a higher number suggests stronger bone structure; trabecular spacing (Tb.Sp, in µm^−1^), in which a smaller number indicates better structure; and trabecular pattern (Tb.Pf, in µm^−1^), which is a measure of connectiveness of trabecular structures and a smaller number indicates more connected bone. Cortical wall thickness for each of the bones was measured manually using a transparency placed over the images, centered on the medullary area of each slice from the 50% region. Eight measurements were collected from the medial, lateral, anterior and posterior planes and the average was calculated to provide one measurement. Whole-bone (whole-femur, -tibia and -vertebrae) measurements of each parameter were calculated using the average of the regional measurements. 

Femur, tibia and spine vertebrae were chosen for analysis because they display significant defects in microarchitecture in humans [1] and mice [49,50,54] experiencing age- and estrogen-deficiency-related bone loss and because they showed significant positive effects of probiotic and polyphenol-rich fruit feeding in previous studies conducted by our laboratory [54,56] and other investigators [37,49,50,57,58]. 

### 2.4. Data Analysis

Statistical power analysis determined that a sample size of six mice per group provided a >80% probability of detecting a significant difference in femur trabecular bone volume/total volume area (a primary outcome measure of the reduction in bone mass in aging mice) [49,59] between the diet groups. 

Data were examined for normal distribution and equality of variances using the Shapiro–Wilk test and analysis of means for variances with Levene test, respectively. Data that were not normally distributed were transformed to log normal prior to analyses. One-way analysis of variance (ANOVA) followed by Tukey–Kramer HSD post-hoc multiple comparisons were used to compare group means when variances were equal. Nonparametric one-way ChiSquare test followed by Wilcoxon nonparametric comparisons for each pair were used when variances were not equal. Descriptive data are reported as means with standard deviations. Percent body weight gain was calculated as: (final (age 16 mo) body weight—initial (age 10 mo) body weight)/initial body weight × 100. Feed efficiency was calculated as (body weight in g/(food intake in g × kcals per g)), using kcals/g for each diet as indicated in Table 1. Final body weight and % body weight gain did not have statistically significant effects on bone outcomes when included as random variables in ANOVAs and thus neither was included in the model. JMP Pro 13 statistical software (Cary, NC, USA) was used to perform data analysis.

## 3. Results

### 3.1. Body Weight and Food Intake

**Figure 1:**
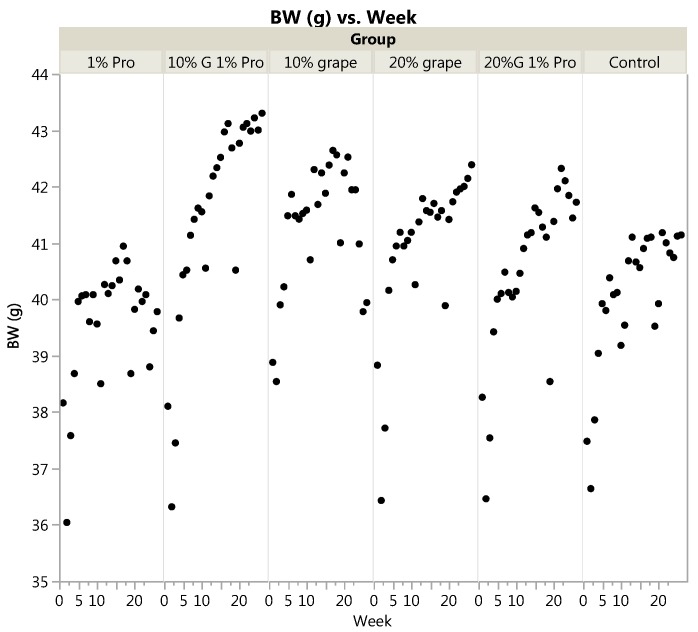
Body weight across weeks by diet group. BW=body weight in g; 1% Pro = 1% probiotic diet; 10% G 1% Pro = 10% grape + 1% probiotic diet; 20% G 1% Pro = 20% grape + 1% probiotic diet.

Initial body weight, final body weight and % change in body weight were not significantly different across diet groups (*p =* 0.99, 0.51, and 0.66, respectively), Table 2. However, mixed models ANOVA with repeated measures showed a significant effect of diet group on body weight across weeks (*p <* 0.0001), with 10% grape + 1% probiotic and 10% and 20% grape groups weighing more than 1% probiotic (*p <* 0.0001, *p* = 0.0002 and *p* = 0.0065, respectively), and 10% grape and 10% grape + 1% probiotic weighing more than control (*p* = 0.0197 and *p* = 0.0042, respectively), Figure 1.

**Figure 2:**
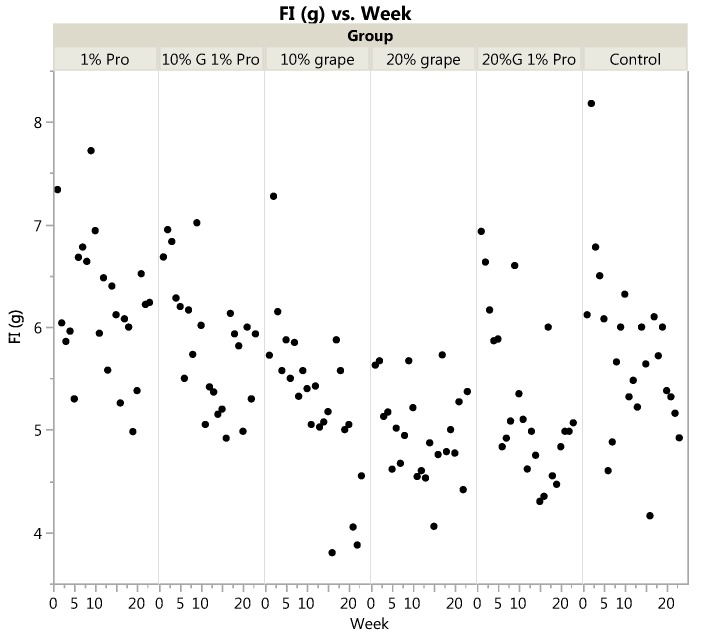
Food intake across weeks by diet group. FI = food intake in g. 1% Pro = 1% probiotic diet; 10% G 1% Pro = 10% grape + 1% probiotic diet; 20% G 1% Pro = 20% grape + 1% probiotic diet. Mixed models ANOVA with repeated measures showed a significant main effect of diet group on food intake (FI, in g) across weeks (*p <* 0.0001). Mice in the 1% probiotic consumed significantly more food per day than mice in the 10% grape, 20% grape, and 20% grape + probiotic groups (*p* = 0.0003, *p <* 0.0001 and *p* = 0.0002, respectively).

Food intake was significantly different across diet groups when analyzed by one-way ANOVA of average intake during the entire experiment and ANOVA with repeated measures across the 24 weeks of the experiment (*p <* 0.0001 for both), Table 2 and Figure 2. Mice in the 1% probiotic consumed significantly more food per day than mice in the 10% grape, 20% grape, and 20% grape + probiotic groups (*p* = 0.0003, *p <* 0.0001 and *p* = 0.0002, respectively). Although mice in the 1% probiotic group ate the most food per day, feed efficiency was significantly less in the 1% probiotic vs. 10% grape, 10% grape + probiotic and 20% grape + probiotic groups (*p <* 0.0001 for all post-hoc comparisons).

Mixed models ANOVA with repeated measures showed a significant main effect of diet group on body weight (BW in g) across weeks (*p <* 0.0001), with 10% grape + 1% probiotic and 10% and 20% grape groups weighing more than 1% probiotic (*p <* 0.0001, *p* = 0.0002 and *p* = 0.0065, respectively), and 10% grape and 10% grape + 1% probiotic weighing more than control (*p* = 0.0197 and *p* = 0.0042, respectively).

### 3.2. Bone Microarchitecture

#### 3.2.1. Tibia

Aging exerted a significant effect on tibia metaphysis trabecular bone, with baseline 10-month-old mice having significantly higher BV/TV and trabecular number measurements and lower trabecular spacing measurements than all 16-month-old groups (*p <* 0.001) (Table 3). Neither grape nor probiotic enrichment improved bone microarchitecture compared to control diet. The combination of 20% grape + 1% probiotic exerted detrimental effects on tibia metaphysis BV/TV compared to 10% grape + 1% probiotic, and trabecular number and trabecular spacing compared to 10% grape + 1% probiotic, 1% probiotic and control groups (*p <* 0.05). Tibia cortical bone showed no significant effects of aging or diet (*p >* 0.05).

#### 3.2.2. Femur

Femur trabecular bone did not demonstrate significant aging effects when comparing 10-month-old mice to 16-month-old mice fed control diet (*p >* 0.05) (Table 4). Neither grape nor probiotic enrichment improved bone microarchitecture compared to control diet. The combination of 20% grape + 1% probiotic tended to produce detrimental effects on femur metaphysis compared to the other diet groups in that BV/TV, trabecular number, trabecular spacing and trabecular pattern factor for 20% grape + 1% probiotic, but not for most of the other diet groups, were significantly different from 10-month-old mice (*p <* 0.05). Femur cortical bone showed no significant effects of aging or diet (*p >* 0.05).

#### 3.2.3. Vertebrae

Vertebrae trabecular bone showed limited effects of aging and diet. The baseline 10-month-old mice displayed the smallest vertebrae trabecular spacing (group main effect *p =* 0.0394) and the largest trabecular thickness (group main effect, *p =* 0.0247; post-hoc analysis baseline vs. control, *p <* 0.05).

## 4. Discussion

The hypothesis tested in this study, that probiotics would enhance bone-protective outcomes of grape-enriched diets, was not supported by these data. Results show that combining probiotics with 10% and 20% grape diets exerted either a null or an interfering effect on bone microarchitecture measures in comparison to independent probiotic and grape dietary enrichment. The underlying rationale for the hypothesis was based on evidence showing that polyphenol-rich diets protect bone from age- and post-menopausal-related loss [37,49,50,57], and that select *lactobacillus* and *bifidobacteria* strains metabolize polyphenols to bioactive metabolites [38,39,40,60,61,62,63,64,65]. Further, prior studies in animals and humans demonstrate that secondary metabolites of polyphenols produced by gut bacteria, including *lactobacillus* and *bifidobacteria* strains, augment the health benefits of polyphenol-rich food consumption [42,43,66,67,68]. Evidence supports that grape consumption protects bone by inhibiting production of inflammatory molecules that stimulate osteoclasts [20,21,22] and by promoting activity of osteoblasts [23,24,27,28]. This mechanism aligns with the concept of age-related bone loss being related to chronic low-grade inflammation and oxidative stress [29,30,31,69,70,71,72]. Zhang et al. [69] showed that biomarkers of oxidative damage to proteins and lipids were significantly higher in plasma and femur from older (18-month-old) vs. young (2-month-old) and adult (6-month-old) rats, and that this age-related increase in oxidative stress was correlated with significantly lower femur bone mineral density (BMD). A negative correlation between lipid peroxidation and femur BMD has also been demonstrated in post-menopausal women with osteoporosis [73]. 

Several studies suggest a beneficial synergism between probiotics and polyphenol-rich diets. Duoeonne et al. [74] described an increase in circulating microbial metabolites of cranberry polyphenols when mice were co-supplemented with the probiotic *Bacillus subtilis* CU1. Hakansson et al. [47] reported improved anti-inflammatory and anti-oxidative biomarker levels in colitic mice fed blueberry husks and rye fiber with *lactobacillus* and *bifidobacteria* probiotics compared to without probiotics. Similarly, reductions in tissue and blood inflammatory markers resulting from a 4% powdered green tea leave diet fed to mice were augmented by co-ingestion of *L. plantarum* [42]. While Jakesevic et al. [43] showed no significant enhancement in anti-oxidative defense following intestinal ischemia-reperfusion in mice when *L. plantarum* was added to a bilberry-enriched diet, their data do not indicate antagonism of the probiotic on bilberry-associated bioactivity. Interestingly, their study demonstrated that adding *L. plantarum* to bilberry- and chokeberry-enriched diets prevented reductions in colonic *lactobacillus* counts caused by the berry diets. The authors suggested that *L. plantarum*, which possesses enzymatic activity toward polyphenols [75,76], may have altered the colonic microbiota profile to facilitate the growth and maintenance of polyphenol-metabolizing *lactobacillus*. Modification of the intestinal microbiota by polyphenols has been documented in multiple human and animal studies as well as in vitro models [46,77,78,79,80,81,82]. For example, *lactobacillus* and *bifidobacteria* concentrations are increased in response to feeding of red wine polyphenols and flavanol-enriched cocoa powder [83,84].

Reasons for the absence of an observed improvement in bone integrity with the combined probiotic + grape diets in this study may include an interaction between the probiotic strains and commensal gut bacteria, leading to impaired production of polyphenol metabolites that are beneficial to bone. Interactions between polyphenols and other dietary components have been previously reported. Blanton et al. [55] described a partial negation of blood pressure reduction in spontaneously hypertensive rats fed blueberry when probiotics were co-ingested. In that study, blueberry-induced increases in urine hippuric acid levels were not impaired by probiotics, suggesting that polyphenol absorption was not blocked by probiotics. However, hippuric acid is one of many metabolites of polyphenols and the production of other bioactive metabolites could have been affected by the added probiotics [85]. Bolca et al. [86] showed that microbial activation of dietary phytoestrogens into bioactive compounds was modified by co-supplementation with different foods. In addition, an interaction between supplemental vitamin C and grape-seed polyphenols was reported by Ward et al. [87], wherein the combination treatment reversed improvements in systolic blood pressure caused by the individual supplements, resulting in an increase in systolic blood pressure in hypertensive patients. 

Another possibility is that a positive effect of grape oligosaccharides and fiber on bone architecture was inhibited by co-ingestion of probiotics. Higher dietary and specifically fruit fiber intake has been shown to protect against femoral neck bone loss in men [88]. Mechanisms include improved mineral solubility and absorption resulting from growth of gut bacteria that ferment oligosaccharides and fiber to short-chain fatty acids [89,90,91]. The growth of commensal gut bacteria that metabolize grape oligosaccharides might have been antagonized by exogenous probiotic bacteria in the grape + probiotic-fed mice in this study. 

A different strategy of using probiotics to metabolize polyphenols to bioactive compounds prior to consumption has also been tested. Lambert et al. [92] demonstrated a significant bone benefit in osteopenic post-menopausal women that consumed probiotic-fermented red clover extract (high in isoflavones) for 12 months. In that study, probiotics were allowed to ferment the extract prior to consumption, whereas the present study was designed to promote grape polyphenol fermentation by probiotics within the mouse intestine. Elsewhere, an equol-rich supplement produced from *lactococcus garvieae*-fermented soy germ showed superior bioavailability to intact soy isoflavones [93]. Consumption of pre-formed polyphenol metabolites thus may be a more reliable method for exploiting probiotic transformation of polyphenols to bioactive compounds than co-ingestion of polyphenol-rich food and probiotics. 

The lack of a significant beneficial effect of the 1% probiotic compared to control diet on bone microarchitecture in this study is not consistent with previous findings and may be related to differences in probiotic strains used. Blanton et al. [56] reported significant protection against femur trabecular loss due to skeletal unloading in adult male rats fed a synbiotic diet consisting of *Lactobacillus acidophilus, Lactococcus lactis lactis* and fructooligosaccharides. McCabe et al. [94] showed significant increases in femoral trabecular parameters in healthy male mice supplemented with *Lactobacillus reuteri* for four weeks. These improved bone outcomes were associated with lower intestinal expression of TNF-α, a marker of inflammation. The investigators also demonstrated that *L. reuteri* supplementation suppressed bone resorption and preserved femur trabecular bone parameters in ovariectomized mice [95]. Using a mouse model of type I diabetes-related bone loss, Zhang et al. [96] showed that four weeks of *L. reuteri* treatment preserved osteoblast function and protected femur trabecular and cortical parameters against loss. Lastly, *Lactobacillus paracasei* and a mixture of *Lactobacillus* strains, including *L*. *plantarum*, prevented femur cortical loss and reduced the expression of femur TNF-α in ovariectomized mice [97]. 

It is important to recognize the differences among animal models of osteoporosis in the site-specificity and rate of bone loss, which might impact treatment effects. Age-related osteoporosis, as modeled in the current study, is characterized by a reduced rate of bone turnover [57,98], whereas ovariectomy is marked by a rapid onset of accelerated bone turnover with an increase in bone resorption and relative deficiency in bone formation [99]. Both models of osteoporosis result in the deterioration of trabecular bone, but differences may exist in cortical bone response. Age-related decreases in cortical bone thickness due to expansion of the medullary region have been reported by some [49,100], but not all [59], investigators. In ovariectomized rodents, cortical bone thickness has been shown to decline [97], increase [101], or remain unchanged [102]. Despite these differences, it is notable that polyphenol-rich fruit consumption has produced beneficial bone responses in both models of osteoporosis [37,49,57,103]. 

The lower body weight, higher food intake, and lower feed efficiency in the 1% probiotic mice are consistent with prior findings from some rodent studies but contrary to those from others, particularly experiments in commercial production animals. McCabe et al. [94] reported a trend toward lower body weight and significantly less visceral fat in mice supplemented with *L. reuteri.* Similarly, Karlsson et al. [104] showed significantly less 6-month weight gain in rats supplemented with *L. plantarum* vs. plain water. Our laboratory previously found no significant effects of synbiotic- or probiotic-containing diets in body weight, food intake, or feed efficiency in male rats [55,56]. A meta-analysis of human and animal studies showed varying effects of different *Lactobacillus* species on weight change, with *L*. *plantarum*, a probiotic used in the current study, promoting weight loss [105]. Probiotic supplementation is commonly used to enhance feed efficiency and body weight gain in commercial animal production [106,107]. 

## 5. Conclusions

In conclusion, dietary co-enrichment with grape powder and probiotics does not produce a synergistic beneficial bone response in aging mice. Moreover, the data suggest an interaction between grape and probiotics that exerts negative bone effects compared to independent dietary enrichment with either supplement. 

## Figures and Tables

**Table 1:** Composition of diets.

Ingredient (g)	10% Grape	20% Grape	10% Grape + 1% Probiotic	20% Grape + 1% Probiotic	1% Probiotic	Control
Casein	140.0	140.0	140.0	140.0	140.0	140.0
Cornstarch	520.7	520.7	510.7	510.7	510.7	520.7
Dextrose	50.0	0.0	50.0	0.0	100.0	100.0
Fructose	50.0	0.0	50.0	0.0	100.0	100.0
Cellulose	50.0	50.0	50.0	50.0	50.0	50.0
Grape Powder	100.0	200.0	100.0	200.0	0.0	0.0
Probiotic	0.0	0.0	10.0	10.0	10.0	0.0
Soybean Oil	40.0	40.0	40.0	40.0	40.0	40.0
Mineral Salt Mix	35.0	35.0	35.0	35.0	35.0	35.0
Vitamin Mix	10.0	10.0	10.0	10.0	10.0	10.0
l-Cystine	1.8	1.8	1.8	1.8	1.8	1.8
Choline Bitartrate	2.5	2.5	2.5	2.5	2.5	2.5
Total g	1000.0	1000.0	1000.0	1000.0	1000.0	1000.0
Total kcals	3762.8	3722.8	3722.8	3682.8	3762.8	3802.8
Kcals/g	3.76	3.72	3.72	3.68	3.76	3.80

Kcal values: Starch, sugars = 4 kcals/g; grape powder = 3.6 kcals/g; casein = 4 kcals/g; oil = 9 kcals/g. Grape component was freeze-dried grape powder made from whole grapes. Probiotic was a freeze-dried powder composed of equal parts *Bifidobacterium bifidum*, *B. breve*, *Lactobacillus casei*, *L. plantarum*, and *L. bulgaricus* at a concentration of 1.0 × 10^11^ cfu/g.

**Table 2:** Body weight, food intake and feed efficiency by diet group.

	10% Grape	20% Grape	10% Grape + 1% Probiotic	20% Grape + 1% Probiotic	1% Probiotic	Control	Main Effect Diet, *p*-Value (One-Way ANOVA)	Main Effect Diet, *p*-Value (ANOVA Repeated Measures *)
Initial Body Weight (g)	37.55	38.82	38.10	38.65	38.16	37.48	0.99	-
	(3.62)	(4.36)	(3.68)	(4.59)	(4.75)	(3.56)		
Final Body Weight (g)	42.20	42.38	43.30	42.16	39.78	41.14	0.51	<0.0001
	(1.97)	(3.87)	(2.06)	(3.48)	(2.55)	(2.82)		
Body Weight Gain (% gain of initial)	12.86	9.59	14.39	9.59	5.10	10.32	0.66	-
	(7.11)	(8.21)	(10.60)	(6.92)	(10.45)	(10.14)		
Food Intake (g/day)	5.29	4.97	5.85	5.27	6.19	5.72	<0.0001	<0.0001 *
	(0.76) ^a,b^	(0.44) ^b^	(0.63) ^b,c^	(0.77) ^a,b^	(0.66) ^c^	(0.82) ^b,c^		
Feed Efficiency (body weight/kcal/day)	2.13	2.22	1.92	2.12	1.72	1.87	<0.0001	<0.0001
	(0.36) ^a,b^	(0.20) ^a^	(0.26) ^b,c^	(0.33) ^a,b^	(0.19) ^c^	(0.30) ^c^		

Values are means (SD). Initial body weight: body weight at age 10 mo; final body weight: body weight at age 16 mo; body weight gain calculated as: (final (age 16 mo) body weight—initial (age 10 mo) body weight)/initial body weight × 100; feed efficiency calculated as: body weight in g/(food intake in g*kcals per g), using kcals/g for each diet as indicated in Table 1. * ANOVA across repeated weeks. Values not sharing letter superscripts are significantly different (*p* < 0.05).

**Table 3:** Tibia microarchitecture in 10-month-old (baseline) mice and 16-month-old mice fed grape- and/or probiotic-enriched or control diet for 6 months.

	10-Month-Old Baseline	10% Grape	20% Grape	10% Grape + 1% Probiotic	20% Grape + 1% Probiotic	1% Probiotic	Control	Main Effect Group, *p*-Value
**Tibia proximal metaphysis**								
BV/TV, %	12.0 (2.1) ^a^	5.9 (3.7) ^b,c^	6.4 (3.3) ^b,c^	6.1 (2.2) ^b^	3.67 (1.8) ^c^	6.1 (1.8) ^b,c^	6.4 (2.7) ^b,c^	0.0007
SA/BV, mm ^−1^	47.047 (3.356)	52.343 (9.674)	48.691 (8.348)	52.942 (3.672)	49.445 (6.944)	48.969 (5.534)	57.136 (6.211)	0.2190
Tb. Th, mm	0.043 (0.003)	0.0392 (0.007)	0.042 (0.007)	0.038 (0.003)	0.041 (0.006)	0.041 (0.004)	0.035 (0.004)	0.2173
Tb. N, mm^−1^	2.817 (0.450) ^a^	1.399 (0.774) ^b,c^	1.482 (0.661) ^b,c^	1.608 (0.512) ^b^	0.862 (0.314) ^c^	1.458 (0.318) ^b^	1.776 (0.558) ^b^	0.0002
Tb. Sp, mm	0.319 (0.050) ^a^	1.345 (1.672) ^b,c^	0.793 (0.424) ^b,c^	0.626 (0.161) ^b^	1.260 (0.497) ^c^	0.676 (0.187) ^b^	0.575 (0.195) ^b^	0.0006
Tb. Patt. Fact, mm^−1^	9.481 (3.098)	17.980 (7.979)	16.242 (8.052)	16.413 (5.642)	18.852 (4.531)	14.671 (5.378)	14.895 (2.887)	0.1611
**Tibia mid- diaphysis**								
Cortical wall thickness, mm	0.263 (0.021)	0.256 (0.015)	0.256 (0.011)	0.258 (0.012)	0.258 (0.017)	0.258 (0.008)	0.260 (0.007)	0.9735
Medullary area, mm^2^	3.585 (0.332)	3.918 (0.288)	3.790 (0.293)	3.630 (0.399)	3.724 (0.278)	4.165 (0.245)	3.919 (0.308)	0.0659

Values are means (SD); *n* = 7 (20% Grape), *n* = 6 (10% Grape + 1% Probiotic and 20% Grape + 1% Probiotic), *n* = 5 (10% Grape, 1% Probiotic and Control). BV/TV, bone volume to total volume fraction; SA/BV, bone surface area to bone volume; Tb.Th, trabecular thickness; Tb. N, trabecular number; Tb. Sp, trabecular spacing; Tb. Patt. Fact, trabecular pattern factor. Values not sharing letter superscripts are significantly different (*p <* 0.05).

**Table 4:** Femur microarchitecture in 10-month-old (baseline) mice and 16-month-old mice fed grape- and/or probiotic-enriched or control diet for 6 mo.

	10-Month-Old Baseline	10% Grape	20% Grape	10% Grape + 1% Probiotic	20% Grape + 1% Probiotic	1% Probiotic	Control	Main Effect Group, *p*-Value
Femur distal metaphysis								
BV/TV, %	11.9 (2.0) ^a^	8.4 (3.4) ^a,b^	8.5 (3.8) ^a,b^	7.6 (3.5) ^a,b^	5.6 (1.7) ^b^	7.4 (2.6) ^a,b^	7.2 (3.7) ^a,b^	0.049
SA/BV, mm^−1^	44.183 (2.633)	40.412 (4.900)	43.594 (3.171)	43.613 (1.917)	45.437 (4.084)	43.105 (7.204)	45.709 (2.784)	0.523
Tb. Th, mm	0.045 (0.003)	0.050 (0.006)	0.046 (0.003)	0.046 (0.002)	0.044 (0.004)	0.048 (0.009)	0.044 (0.003)	0.473
Tb. N, mm^−1^	2.622 (0.412) ^a^	1.641 (0.593) ^a,b^	1.821 (0.720) ^a,b^	1.645 (0.735) ^a,b^	1.266 (0.429) ^b^	1.572 (0.424) ^a,b^	1.605 (0.735) ^a,b^	0.018
Tb. Sp, mm	0.344 (0.061) ^a^	0.678 (0.431) ^b^	0.614 (0.342) ^a,b^	1.072 (0.864) ^b^	0.802 (0.201) ^b^	0.628 (0.188) ^b^	0.667 (0.299) ^a,b^	0.052
Tb. Patt. Fact, mm^−1^	12.439 (3.132) ^a^	12.981 (3.031) ^a,b^	14.433 (3.293) ^a,b^	15.562 (1.944) ^a,b^	17.863 (1.650) ^b^	15.143 (3.135) ^a,b^	16.261 (2.360) ^a,b^	0.0373
Femur mid- diaphysis								
Cortical wall thickness, mm	0.254 (0.027)	0.264 (0.031)	0.264 (0.015)	0.252 (0.019)	0.252 (0.017)	0.279 (0.034)	0.258 (0.010)	0.4509
Medullary area, mm^2^	6.081 (0.514)	6.807 (0.534)	6.387 (0.583)	6.653 (0.319)	6.641 (0.429)	6.807 (0.551)	6.478 (0.097)	0.1659

Values are means (SD), *n* = 7 (20% Grape), *n* = 6 (10% Grape + 1% Probiotic and 20% Grape + 1% Probiotic), *n* = 5 (10% Grape and 1% Probiotic), *n* = 3 (Control). Values not sharing letter superscripts are significantly different (*p <* 0.05).
